# Lime Water in Infantile Eczema

**Published:** 1876-04

**Authors:** 


Lime-Water in Infantile Eczema.—A writer in the *Bulletin de Therapeutique* recommonds lime-water in eczema of the head and impetigo of the face in children, especially chronic cases which have resisted other treatment, and states that a marked improvement is noticeable after using it for eight days. It is to be taken in quantities varying up to half a pint, according to the age of the patient, and to dust the part with carbonate of magnesia; but the latter is only necessary when the secretion is very irritant.—*Medical Brief.*

